# Measuring access to medicines: a review of quantitative methods used in household surveys

**DOI:** 10.1186/1472-6963-10-146

**Published:** 2010-05-30

**Authors:** Vera Maria V Paniz, Anaclaudia G Fassa, Maria de Fátima S Maia, Marlos R Domingues, Andréa D Bertoldi

**Affiliations:** 1 Programa de Pós-graduação em Epidemiologia, Universidade Federal de Pelotas, Pelotas, Brazil; 2 Programa de Pós-graduação em Saúde Coletiva, Universidade do Vale do Rio dos Sinos, São Leopoldo, Brazil; 3 Programa de Pós-Graduação em Educação Física, Universidade Federal de Pelotas, Pelotas, Brazil

## Abstract

**Background:**

Medicine access is an important goal of medicine policy; however the evaluation of medicine access is a subject under conceptual and methodological development. The aim of this study was to describe quantitative methodologies to measure medicine access on household level, access expressed as paid or unpaid medicine acquisition.

**Methods:**

Searches were carried out in electronic databases and health institutional sites; within references from retrieved papers and by contacting authors.

**Results:**

Nine papers were located. The methodologies of the studies presented differences in the recall period, recruitment of subjects and medicine access characterization.

**Conclusions:**

The standardization of medicine access indicators and the definition of appropriate recall periods are required to evaluate different medicines and access dimensions, improving studies comparison. Besides, specific keywords must be established to allow future literature reviews about this topic.

## Background

Universal medicine access is a major goal of the World Health Organization (WHO) and of most countries with respect to medicine policy[1]. This goal is included in the objectives of the Millennium Development [2,3]. The WHO defines medicines access as the equitable availability and affordability of essential medicines during the process of medicine acquisition [4]

The global situation of access to essential medicines is still considered critical. Although the access to health care is a fundamental human right and includes essential medicine access, the World Health Organization (WHO) estimates that nearly two billion people (1/3 of world population) are not receiving regularly all medicines they need. In developing countries the budget for medicines corresponds to 24-66% of national health expenses, supporting the monitoring of access to medicines in these countries [3].

Nearly ten years ago the WHO developed a set of indicators to evaluate drug policies[5]. Recently, the WHO and the Management Science for Health (MSH) established indicators and methods to evaluate pharmaceutical assistance on three levels [5-8]. The level I evaluates structure and process; level II evaluates results and; level III evaluates each country's specific aspects. Within the level II, methods to assess household-level medicines use and access has already been tested in some countries, including Brazil [1].

Household surveys are crucial to provide information on how drug policies affect the individual's well being, as this kind of study allows to evaluate access from the perspective of the individual, regardless of his/her use of health services. This is especially relevant when evaluating medicine access to drugs prescribed for chronic conditions, as these drugs may not be linked to a recent visit to the doctor. However, household surveys are expensive and logistically complex. The methodology suggested by WHO and MSH evaluates if and how people are obtaining their medicines, including how they use them, how much is paid and what is the burden of this payment on the overall family income [6].

Nonetheless, up to now there is no operational definition of medicine access, not even a model to monitor medicine access in a country or to make comparisons across countries [9]. Therefore, this review describes quantitative methodologies to measure medicine access and explains how access is characterized. The advantages and disadvantages of different methodologies are described. The goal of the paper is to contribute to the standardization of methodologies, increasing studies comparability.

## Methods

A literature review was performed in electronic databases, health institutions websites, references of papers retrieved and contact with authors. Electronic databases were: PubMed, Lilacs, Embase, Web of Science and the Scientific Electronic Library Online (SCIELO). The institutional websites were: the World Health Organization http://www.who.int; Brazilian National School of Public Health http://www.ensp.fiocruz.br, Brazilian Health Ministry http://www.saude.gov.br, Pan American Health Organization http://www.paho.org and the Management Sciences for Health http://www.msh.org/seam.

With respect to the search, no limits were used and keywords appearing on any field of the paper were accepted. This strategy was chosen because recent papers are more likely to be traced by title and/or abstract words, for example, *ahead of print *papers.

The search was carried out using the following keywords: *drug utilization; drug access; drug use habits; essential drugs; drug prescription; drugs expenditures; drug delivery systems; drug evaluation; self-medication; pharmacoepidemiology; pharmaceutical preparations*; *medication; medicine*. As the words 'medicine' and 'drugs' are highly cited in medical literature, many times as illegal drugs, during search these words were combined with other terms to make results more precise. The chosen terms were: *primary care; patient care; health care; primary health care; family health program; aging health; health services; health services accessibility; hypertension; diabetes mellitus; compliance; prescription; knowledge, attitudes, practice; risk factors; aged; elderly*.

Quantitative studies that measured medicine access on household level as the main or secondary outcome and were published until July 2008 were included in the analysis. The access was defined as obtaining the medicine for free or by payment [10]. Thus, studies about accessibility (geographical access), adequacy (appropriateness of medicine prescribing) and medicine acceptability by the patient were not included in the review.

The following exclusion criteria were employed: review papers; qualitative design studies (once the methodology do measure access is specific and not comparable to quantitative studies); studies with data collection that were not household-based interviews (this means that demand studies or studies made in a health facilities were excluded and the subjects in the study are not necessarily users of health services); studies that, although home-based, only measured affordability (health expenses), and did not fulfill medicine access criterion for this review; specific papers about medicine access for treatments such as STD/AIDS (since each country has its own legislation for distribution of these drugs), tuberculosis, Hansen's disease and endemic diseases control (malaria, leishmaniasis, schistosomiasis, etc); studies about access to high cost medicines prescribed for complex health problems; studies with people covered by health insurance plans (Medicare, Remediar), as they differ from country to country depending on the medicine supply methods; and studies on specific ethnic groups.

**Figure 1:**
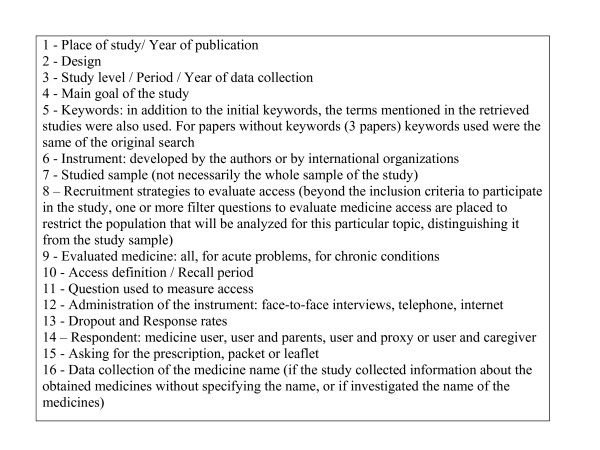
**Information obtained from the selected studies**.

A box is presented at the end of the text showing information retrieved from the selected papers. These data were summarized to identify disadvantages and advantages of employed methodologies (Figure 1).

## Results

Using mainly the keyword medicine use [words]/drug utilization [any field], more than 9000 titles and/or abstracts were retrieved until July, 2008. After reading titles/abstracts, nearly 8750 papers were excluded because data collection was not home based. The papers were mostly about demand, medicine availability, in the public/private sector, medicine pricing/expenditure in the public/private sector, medicine prescription studies, health insurance coverage (based on secondary data), co-payments impact or qualitative studies. From the remaining papers, around 180 were excluded because did not evaluate access or were review papers. In about 70 papers we could not identify by the abstract if the approach was the desired (home-based data collection about access), and in those cases full texts were read and even if medicine access was not the main outcome the study could be included.

The references of selected studies were checked to find other articles that could be included in the review. Besides, author searches were carried out using relevant names in the field. When more than one study was originated from the same data collection, only one study was included in the review.

After manual search, impaired by the lack of specific keywords to evaluate medicine access, nine studies were included in this review [1,11-18].

In Additional file 1 it can be observed that among selected studies, six were conducted in South America [1,11-15], two in North America [16,17] and one in Asia [18], most were published after 2004. All studies were cross-sectional [1,11-18], nearly half of the studies were national [1,12,16,17] two were regional [15,18] and three were municipal [11,13,14] and in only one study data collection took place before the year 2000 [14].

Medicine access was the main outcome of six out of nine presented studies [1,11,15-18]. For the remainder [12-14], evaluation of access was done by adherence studies [13] and medicines use [12,14]. With regard to keywords, "drug utilization" was the most used (six studies) [11,12,14-17] while only two studies included "access" [11,17], although the term was not referring to medicines, but to health services accessibility (Additional file 1).

Nearly half of the studies assessed the outcome by instruments developed by the researchers [11,15,16,18]. Two studies utilized the methodology suggested by WHO [1,12], and only aimed at evaluating medicine access [1], the other was designed to evaluate health services performance [12] (Additional file 1).

Additional file 2 presents population and assessed medicines, as well as the access definition used on each study. As for the sample, six were population-based studies [1,12-14,16,17], two studied the population covered by Primary Care Unit [11,15] and one investigated local health services users [18]. All studies evaluated medicine access including the elderly [1,11-18], one third of studies included all age groups [1,11,14] and three studied people who were 18 or older [12,17,18]. The smallest sample size was 248 people (all ages) [14], but most studies [1,11-13,15-17] counted on sample sizes larger than 2000 people.

The criterion employed to include subjects in the specific medicine access evaluation was not similar between studies. Some studies recruited individuals based on the need for the prescribed medicine [13,15,17], one study was specific to three chronic conditions [15] and another included subjects facing cost pressures [17]. Four studies linked evaluation of the access to medicines prescribed in the last visit to the doctor [1,12,14,18]; one study excluded chronic conditions consultations [1]. From the remaining two studies, one did not restrict sampling to evaluate access, relying on respondent's report of medicine need [11] and in another study, respondents who reported that they used prescription medication for at least one of the five chronic illnesses were asked about access [16] (Additional file 2)

In terms of assessed medicines, six studies investigated all medicines types [11-14,17,18], one evaluated only medicines prescribed to acute health problems [1] and two other studies included only medicines used to treat chronic conditions [15,16] (Additional file 2).

With respect to the definition of access, the studies presented distinct approaches and distinct recall periods, from 15 days to 12 months. Two studies defined access as obtaining medicines that were prescribed in the last visit to the doctor during the 15 [1] or 30 [14] days prior to the interview, while two other referred only to the last visit with no mention of recall period [12,18]. One study defined lack of medicine access when the person needed but did not use the medicine in the last 15 days, regardless of medical prescription [11]. In two studies, access was defined as obtaining medicines indicated by a doctor, during a 30-day period [15] or 12-month period [17], and only the latter discussed acquisition considering affordability. However, in two studies [13,16], access was defined using a very different criterion. One defined access as adherence [13], that is, individuals that did not interrupt medicine use in the last 12 months, regardless of indication. In the other study [16], underuse was the definition to measure access in a 30-day or 12-month period for medicines prescribed to treat chronic conditions (Additional file 2).

Additional file 3 describes the questions used to evaluate medicine access and logistical aspects related to administering the instruments of the studies included in the review. Only one study did not present the question used to evaluate medicines access [14]. Three studies included the terminology "fail to use/take the medicine" in the question [11,13,15], while four studies used the terms "obtaining of the medicines prescribed/needed" [1,12,17,18]. In two studies the access question referred to affordability [16,17] and in two other studies the terminology "took less medication than prescribed" was employed [13,16].

From all studies, only one relied on internet information (self-administered questionnaire) [16], eight studies collected data by face-to-face interviews [1,11-15,17,18] and one was by phone interview [17]. Three studies presented response rate below 85% [13,16,17]. Some papers do not present clearly who was providing information during interview/data collection [12,16-18] among the studies that state who was the respondent, information was provided by the user [1,11,13,15], by parents (studies with children) [11] and by proxy or caregiver when elderly subjects were studied [1,13]. Only one study used information from the family head [14]. Asking for the packet of the medicine was a strategy of most studies [1,11-13,15] that collected data face-to-face and in two studies the methodology is not entirely described [14,18]. Only three studies adopted the strategy of asking for the medicine prescriptions [11,12,15]. Besides evaluating medicine access, five studies [1,11-13,15] also collected the name of prescribed medicines, presented or reported by the user, while the rest of studies did not present such information [14,16-18] (Additional file 3).

## Discussion

The acknowledgment of methodologies available to evaluate medicine access on household level is the first step to establish strategies that will increase the understanding of individual barriers to medicine access. However, when summarizing methodologies, the first obstacle is the search itself that demanded different strategies and intense manual work. The literature about medicine access on household level is sparse. Besides, the available studies relied on distinct methodologies, impairing comparison. The following is a discussion of obstacles to the review, and adjustments to methodological approaches that could allow future comparisons. Although our intention was not to evaluate or to suggest new sampling methods to measure medicine access, adequate sampling techniques and high response rates are fundamental methodological aspects to assure precision and validity in any quantitative study.

## Obstacles to the review

### Keywords

The terms "medicine access" are not recognized as keywords, making the search about this topic even more complicated. The search for studies about medicine access on household level results in many health services and/or medicine access/use studies. However, chronic users (chronic diseases medicines) or regular users (oral contraceptives) may have medical indications to use drugs without recent or regular visits to a doctor. Besides, some medicines sold over the counter (antithermic medicines) are not evaluated by this approach. Therefore, a specific keyword to describe medicine access is needed, to detect studies about this theme avoiding manual searches.

### Main goal of the study

The study of medicine access as a secondary goal [12-14] may result in lower level of detail about this aspect and in greatest difficulties in the bibliographic search. Usually, medicine access is included in studies aiming to evaluate medicines use. Many abstracts do not state clearly if the study evaluated medicine access. Place and means of acquisition (paid out-of-pocket/free of charge) are more often mentioned. Thus, whenever utilization or service indicators are mentioned, a complete reading of the paper to check if medicine access was brought up is needed, demanding exhaustive manual work.

### Access definition

One of the biggest obstacles to gather studies about this topic is the diversity of indicators and dimensions used in different definitions of the outcome, with no agreement between studies. Even though during selection of the studies for this review, a single definition was adopted - *"obtaining the needed medicine" *[10], an attempt to exclude studies that evaluated different access dimensions such as availability, geographic accessibility and accommodation [9], we observed that such definition may include not only the medicine acquisition but also the cost-related underuse [16], to evaluate if the individual cut back on treatment due to financial restrictions. Medicine obtaining is represented by the affirmative that the individual did not restrict medication or obtained all prescribed medicines. On the other hand, underuse seems to be another access definition, indicating that the individual cut back on treatment or took less medicine than prescribed. However, this approach (underuse) must be taken cautiously as it could be understood simply as not using medicine for any other reason and not lack of access, if it is not clearly stated that the underuse is due to cost.

Medicine adherence was the definition of access used in one study [13], considering that if the individual took less or did not take the prescribed medicine at all, there was no adherence. However, if the reason for not taking the medicine was the lack of affordability, the non-adherence was actually lack of access to the medicine. Thus, the terminology used in such studies, like use, access, adherence and rational use, must be clearly defined.

### Place of the study/Year of publication

This review did not include studies about medicine access for treatment of STD/AIDS and those designed to evaluate only people covered by health insurances - most studies carried out in the US. We also restricted the search to home-based data collections. This strategy may explain the high number of South American publications retrieved.

Three studies carried out in Brazil belonged to the World Health Survey, a multicenter (71 countries); to the SABE project (Health Well-being and Aging), a study that took place in six Latin American countries and The Caribbean; and to the National Pharmaceutical Evaluation, using the methodology suggested by the World Health Organization for all countries[1,12,13]. We could not find publications from the other countries about medicine access referring to this project. However, this is not a limitation of the review, because, as all studies had to use the same methodology, only one study would be included.

The increase in publications after 2004 may indicate the concern of researchers with the monitoring of medicines national policies, attempting to reach the goals of WHO for the period 2004/2007 in response to the challenges in essential medicine access [3].

## Methodological aspects of access evaluation

### Study Level

Studies on national, state, regional or city level result in different conclusions about medicine access. Distinct levels may evaluate either national drug policies assistance or specific national, regional or local programs [19]. Broader studies provide extensive information about access patterns to healthcare services, useful information for policy makers, while smaller and local studies should find answers to specific problems and help the management of local health services. However, this aspect is directly connected to the research question and, consequently, to the instrument that will be used or developed.

### Instrument

The lack of consistency between instruments used to evaluate medicine access and the large variability of approaches poses difficulties to compare the studies and confirms the subject as an area under major conceptual and methodological building [20].

Most studies recruited individuals using as starting point a visit to the doctor (during a certain period) and investigated if the prescribed medicine was obtained. Therefore, some instruments reveal more about health services access than medicine access [8]. This kind of instrument is adequate to evaluate medicines prescribed for high-prevalent acute conditions, but impairs the evaluation of medicine access for chronic diseases, since under this circumstance the visit to the doctor may have happened long before the adopted recall period [9] and does not detect the access to medicines that are not dependant on prescription.

Thus, the choice for an instrument to study this topic must consider the population type (general population, service users, people living in the surrounds of primary care units); the age group to be evaluated (all age groups, only children, only adults, elderly people); and the kind of medicine that will be evaluated (acute problems, chronic problems, all kinds), only then the recall period and recruitment methodology can be established. Those aspects will influence the structure of the question(s) about access; the chosen strategy to administer the questionnaire (face-to-face or self administered); and the respondent definition (user, parents, caregiver, proxy or family head). The quality of information will depend on such characteristics and on the dropout rate of the study.

### Population

The kind of sample to be studied (general population, users or people living in areas covered by primary care units) will determine the kind of question about access to be included in the study. When the general population is interviewed, the study can describe how medicines are obtained by all socioeconomic groups. When studying public health services users and/or when the population belongs to low socioeconomic groups, the main aspect to be investigated is medicine free supply.

In addition to the kind of sample to be studied, age groups must be considered as well. Many studies evaluated all age groups and all included the elderly population. Elderly people present high prevalence of chronic conditions and constantly need to take more medicines continuously, while children and adolescents mostly need medicines to treat acute health problems.

### Types of medicines

A great limitation found in the reviewed studies is the use of the same methodology to investigate access to different types of medicines (prescribed for chronic/acute diseases and even not prescribed), hindering the quality of information. The evaluation of medicine access to treat chronic or acute conditions must rely on different recall periods. Besides, during the planning of the study, the goals of the research must be clear to define the inclusion or not of over the counter medicines, adapting the question for this definition. When asking if the individual restrict medication during a certain period, over the counter medicines will be included as well.

### Recall Period

In studies about medicine access recall bias is a potential source of error; respondents may recall better information of the medicines they had access to. In an attempt to control such problem, the recall period to be used in medicine access studies depends on the medicine access characterization, on the type of medicine to be evaluated and on the studied age group.

As described previously, each medicine access definition considers different aspects. The access, defined as obtaining prescription drugs, is employed to investigate prescriptions from the last visit to the doctor during a certain period (15-30 days), which is useful to measure medicine access to acute health problems, and also to evaluate chronic use of long-term medicines (12 months), as long as restricted to the medicine type for specific conditions. On the other hand, the underuse showed by one of the studies [16], was based on a 12-month recall period to evaluate cost-related lack of access to medicine prescribed for chronic illnesses.

Usually, to measure medicine access for acute problems, a short recall period can be used and for chronic conditions longer periods may be indicated to evaluate problems affording prescriptions to continue treatment. Therefore, in studies investigating medicine access for the treatment of chronic and acute health problems, the adoption of distinct recall periods must be considered.

### Recruitment strategies to evaluate access

Often before measuring access it is necessary to define inclusion criteria or use filter questions in order to allow or restrict the type of medicine evaluated. Medicine access studies are usually carried out within studies about health services utilization because there is a need for a diagnosis and consequent prescription. Because of that, many times the recruitment of individuals for the study takes place after the diagnosis of a specific illness and treatment prescription or after a doctor visit that resulted in a prescription. Another approach is to ask about medicine use to treat specific diseases or to consult a list of health service/facility users that attended the service during a specific period. Consequently, the whole population investigated by such studies may be much larger than the sample considered during the evaluation of medicine access.

### Question to evaluate access

The questions to evaluate access focus mainly on two aspects: medicine use/acquisition and underuse (due to costs). According to the indicators suggested by WHO, these evaluation strategies may actually measure the following access dimensions: availability, affordability or both [1]. The questions that investigate if the individual did not use/obtained the prescribed/needed medicine allow two possibilities: if affirmative, the reason can be investigated and, if negative, the information about means of acquisition (paid or free) can be obtained - evaluating both dimensions. When lack of access due to cost is investigated, other reasons are not assessed, restricting evaluation to affordability.

### Instrument administration, dropouts and prescription, packet or leaflet requesting

Most studies had large samples. On the other hand, the two studies based on phone or internet interviews presented high refusal rates [16,17]. Despite such strategies allow studying larger samples, the low response rate is a major limitation. Besides, distant inquiry methods do not allow asking for packets or prescriptions, impairing medicines identification. The packets or prescriptions increase the accuracy and completeness of the information obtained, minimizing memory bias. Medicine access may be overestimated by the bias, since people recall better medicine that they could obtain.

### Name of the medicine

When the name of medicine is collected two different analysis are possible: the first uses as denominator the individuals and evaluates the amount of medicines obtained compared to the needed number categorizing the result as total, partial or no access; the second uses the number of medicines as denominator and measures access to each pharmacologic group or chemical name of the medicine. Even incomplete information can be used, for example, if there is report of a medicine prescription for a specific condition but the name of the medicine is not available, the questions about medicine access can still be answered or the reason for the lack of access, linking these data to the condition that the medicine was prescribed for.

On household surveys, to collect the name of the medicine demands good training of the staff, considering that interviewees usually are not able to inform it accurately. When this is the case, to ask for the medicine packaging or prescription and use help from other informers aware of medicine regimen may increase the quality of data.

### Respondents

When study subjects are children or elderly people, usually they are not aware of the details of medicine use and access. For children, one of the parents needs to provide the information while among the elderly usually a proxy or a person in charge of care is interviewed. Some reviewed studies do not allow identifying the respondent; however this information is extremely important to evaluate the accuracy of information. Another group of studies excluded individuals unable to respond to the questionnaire, and these individuals may need to take regularly many medicines, presenting higher risk of access barriers.

## Conclusions

This review summarized methodologies employed to evaluate medicine access on household level. In spite of the increasing number of publications in the last five years, due to the relevance of the theme, it is surprising that such small number of studies was detected.

This paper revealed the variety of methodologies to evaluate medicine access with respect to recall period, sampling and characterization of the outcome - medicine access.

Therefore, the standardization of medicine access indicators such as acquisition, expenses and categorization according to the dimension of evaluation (availability, affordability, geographic accessibility, acceptability and accommodation) is needed, as well as the establishment of the appropriate recall period to assess each medicine type and each access dimension, increasing comparability across studies. Thus, as there is no standard methods to evaluate household-level medicine access, as defined previously in this review, we propose studies targeted on population access to all medicines, regardless of consumption pattern (chronic or acute). Although both consumption patterns are assessed together, specific questions must be employed for on each situation. For acute problems, a 15-day recall is appropriate, linking the question to the acute health problem (including self-medication) or to the medical appointment within this period (specific to the medical indication) always asking for the package and prescription, to obtain the medicine's name accurately. With respect to chronic problems, a longer recall period (from 30 days to twelve months) would be appropriate to investigate underuse of the medicine and the reasons for it. On those situations, the question must be linked to the disease and package and prescription should be used as well. However, on both approaches the goals of the study, population and age groups to be studied must be established, to define the respondent of the interview. Besides, specific keywords must be used to allow future studies about medicine access.

## Competing interests

The authors declare that they have no competing interests.

## Authors' contributions

**VMVP **worked on the project, literature review and writing of the paper. **AGF **worked on the project and writing of the paper. **ADB **worked on the project and writing of the paper. **MFSM **worked on the literature review. **MRD **worked on the writing of the paper and translation into English. All authors were involved in the final review of the manuscript and all authors read and approved the final manuscript.

## Pre-publication history

The pre-publication history for this paper can be accessed here:

http://www.biomedcentral.com/1472-6963/10/146/prepub

## Supplementary Material

Additional file 1 **Table 1**. Description of studies measuring medicines access on household level. Click here for file

Additional file 2 **Table 2**. Population/medicines studied and definition of medicines access on household level. Click here for file

Additional file 3 **Table 3**. Question/Logistics of instrument's administration in studies evaluating medicines access on household level. Click here for file
