# Supplementary material for: Measuring access to medicines: a review of quantitative methods used in household surveys
Source: BMC Health Serv Res. 2010 May 30;10:146. doi: 10.1186/1472-6963-10-146 (PMC2890644; doi:10.1186/1472-6963-10-146)
Supplement: Additional file 1 — Table 1. Description of studies measuring medicines access on household level. [file 1472-6963-10-146-S1.DOC]

**Table 1-** Description of studies measuring medicines access on household level.

| **Author /Study Setting /**  **Year / Design** | **Study Level /**  **period/year** | **Main goal of the study** | **Keywords** | **Instrument** |
| --- | --- | --- | --- | --- |
| Bertoldi et al. /  Brazil, 2008  Cross-sectional | Southern City /  July-September / 2003 | To describe medicine utilization patterns, to evaluate access and free medicines access | *health care quality, access, and evaluation; drug utilization; health expenditures; pharmaceutical policy; developing countries* | Developed by the authors |
| Paniz et al. /  Brazil / 2008 /  Cross-sectional | South and Northeast regions/  March-August / 2005 | To evaluate the prevalence of access to continuous-use medicines and associated factors | *drug chronic use;*  *drug utilization; hypertension; diabetes mellitus; mental health* | Developed by the authors |
| Tediosi et al. /  Tajikistan / 2008 /  Cross-sectional | Two rural districts /  May-June / 2005 | To evaluate patient expenditures for primary care services  and accessibility of prescription medicines in two rural  districts served by family medicine | *Prescriptions, Drug*  *AND*  *primary health care ** | Developed by the authors |
| Carvalho MFC/  Brazil / 2007  Cross-sectional | Southeastern City/  2000 | To study the risks of polimedication among elderly people from Sao Paulo, Brazil | *Elderly; pharmacoepidemiology; medicine, inappropriate use; survey* | SABE – developed by the researchers of the multicenter study, coordinated by PAHO  (Latin America/ Caribbean) / |
| Carvalho et al. /  Brazil / 2005 /  Cross-sectional | National /  January-September / 2003 | To describe medicine utilization in the Brazilian population | *drugs;*  *drug utilization;*  *drug prescriptions* | WHO to evaluate health systems performance of the member countries  (adapted to Brazil) |
| PAHO, WHO, MH /  Brazil /2005 /  Cross-sectional | National /  September / 2004 | To evaluate the results of pharmaceutical policies with respect to access, quality and rational medicines use | *Health policy; pharmaceutical assistance; medicine policy evaluation;*  *sanitary vigilance; Brazil* | WHO for the household survey about medicines access/use (adapted for Brazil) |
| Reed M. /  United States/ 2005  Cross-sectional | National /  2000-01 and 2003 | To investigate the proportion of adults facing problems to obtain prescribed medicine | ***Drugs; medication; medicine; drug utilization AND***  ***health services accessibility **** | Center for Studying Health System Change (HSC) Community Tracking Study (CTS)  Household Survey |
| Piette et al. /  United States/ 2004  Cross-sectional | National /  November–December/ 2002 | To measure cost-related medicine underuse, population at risk, treatments people forgo, and how often | *Drug utilization ** | Developed by the authors |
| Fernandes MEP /  Brazil / 1998  Cross-sectional | City from the Northeast region /  February-May/ 1997 | To measure medicine utilization with respect to health expenses | *Health assistance; health policy; health economics; medicine use; medicine expenses* | Based on the UNICEF document  – The Bamako Initiative –  “Household survey on medical attention and health expenses on household level” |

*Keyword used during paper search
