# Supplementary material for: Measuring access to medicines: a review of quantitative methods used in household surveys
Source: BMC Health Serv Res. 2010 May 30;10:146. doi: 10.1186/1472-6963-10-146 (PMC2890644; doi:10.1186/1472-6963-10-146)
Supplement: Additional file 2 — Table 2. Population/medicines studied and definition of medicines access on household level. [file 1472-6963-10-146-S2.DOC]

**Table 2-** Population/medicines studied and definition of medicines access on household level.

| **Author / Year** | **Studied sample** | **Recruitment strategy**  **to evaluate access** | **Evaluated Medicine** | **Access definition/ Recall period** |
| --- | --- | --- | --- | --- |
| Bertoldi et al. / 2008 | 900 households,  2988 individuals of all ages,  living in an area covered by PCU* | Same inclusion criterion | All | Lack of access= prescribed medicine, not taken in the last 15 days  Lack of free access= paid medicine taken in the last 15 days |
| Paniz et al. /  2008 | 1244 adults (30-64 years) and 2706 elderly (65 years or older), living in areas covered by PCU* from 41 cities with more than 100 thousand inhabitants in seven states of two Brazilian regions. | Diagnosis of one of the three conditions ** and need for chronic medicine use to treat the conditions | For hypertension, diabetes and mental health | Total, partial or no access = obtaining all, some or none of the needed medicines in the last 30 days |
| Tediosi et al. / 2008 | 901 patients aged 18 years or above, users of primary health care facilities from two districts, in April, 2005 | Having used health service and being prescribed any medicine | All | Obtained all medicines prescribed by the family doctor in the last visit |
| Carvalho MFC/ 2007 | 2143 people, 60 years or older, living in the urban area of Sao Paulo city, in 2000 | To be in treatment with prescribed medicine | All | Adherence = individuals that did not interrupt medicine use in the last 12 months |
| Carvalho et al. / 2005 | 5000 adults, 18 years or older, 5 thousand households, 250 census tracts,  188 cities, 25 estates | Last visit to the doctor resulting in prescription | All | Access to all medicines prescribed in the last visit; obtained most of medicines; obtained fewer medicines; no access |
| OPAS, OMS, MS  2005 | Single-resident households, of all ages, acute illness in the last two weeks, in 916 houses, five states, two cities each state | Being diagnosed with an acute illness in the last 15 days, visiting a doctor and being prescribed any medicine | For acute conditions | Access to all medicines prescribed in the last visit; obtained fewer medicines; no access |
| Reed M. /  2005 | 46400 (2001) and 36500 (2003), working-age population (18-64) | To be in treatment with prescribed medicine | All | No problems to obtain all medicines prescribed in the last 12 months |
| Piette et al. /  2004 | 4055 members of the study panel (1999), interviewed in 2002, 50 years or older,  taking medicines for one of the five listed chronic conditions *** | To be taking medicine for one of the five chronic conditions *** | Prescribed for chronic illnesses | 1-treatment-specific underuse for each medication; 2-some underuse at least once a month;  3-underuse / last year; 4-underuse for one or more medications once a month |
| Fernandes MEP / 1998 | 248 people of all ages, visiting doctors in the last 30 days, 58 census tracts from 29 areas of Fortaleza, Brazil | Having used health service and being prescribed any medicine | All | Provision at health facility of all, some or none medicine prescribed in the last 30 days or paid for the medicines |

* Primary Care Unit

** Hypertension, Diabetes Mellitus, Mental Health Problems

*** Diabetes mellitus, depression, heart problems, hypertension or high cholesterol
