# Supplementary material for: Measuring access to medicines: a review of quantitative methods used in household surveys
Source: BMC Health Serv Res. 2010 May 30;10:146. doi: 10.1186/1472-6963-10-146 (PMC2890644; doi:10.1186/1472-6963-10-146)
Supplement: Additional file 3 — Table 3. Question/Logistics of instrument's administration in studies evaluating medicines access on household level. [file 1472-6963-10-146-S3.DOC]

**Table 3-** Question / Logistics of instrument’s administration in studies evaluating medicines access on household level.

| **Author / Year** | **Question used to measure access** | **Administration of instrument** | **Dropout - Response rates** | **Respondent** | **Asking for the packet, leaflet or prescription** | **Collection of information about name of medicine** |
| --- | --- | --- | --- | --- | --- | --- |
| Bertoldi et al. / 2008 | *“In the last 15 days, did you take all the medicines you needed?”* | Administered by interviewer | 4.4% | User and parents | Packet and prescription | Yes |
| Paniz et al. /  2008 | *“In the last month, did you take all the medicines you needed?”* | Administered by interviewer | Below 5% | User | Packet, leaflet or prescription | Yes |
| Tediosi et al. / 2008 | *“Did you obtain the medicines prescribed by the Family Doctor?”* | Administered by interviewer | 9.9% | Not informed | Not informed | Not informed |
| Carvalho MFC/ 2007 | *“In the past 12 months, have you ever taken less of this medication than prescribed by your doctor?”* | Administered by interviewer | Response rate = 84.6% | User and proxy | Packet | Yes |
| Carvalho et al. / 2005 | *“In your last visit to the doctor, did you get any prescription? If yes, did you get all medicines?* | Administered by interviewer | None declared | Not informed | Packet and prescription | Yes |
| OPAS, OMS, MS  2005 | *“Did you get all medicine prescribed by the doctor/dentist?”* | Administered by interviewer | None declared | User and caregiver | Packet | Yes |
| Reed M. /  2005 | *“During the past 12 months, was there any time you needed prescription medicines but didn’t get them because you couldn’t afford it?”* | Phone interview | Response rate in 2001= 59% Response rate in 2003= 57% | Not informed | Non-applicable | Not informed |
| Piette et al. /  2004 | *“In the past 12 months, have you ever taken less of this medication than prescribed by your doctor because of the cost?”* | Self-administered (internet) | Response rate =76% | Not informed | Non-applicable | Not informed |
| Fernandes MEP / 1998 | *Not informed* | Administered by interviewer | None declared | Family head | Not informed | Not informed |
